# Efficacy and safety of neoadjuvant immune checkpoint inhibitors combined with chemotherapy in locally advanced hypopharyngeal squamous cell carcinoma: a retrospective analysis

**DOI:** 10.3389/fonc.2026.1928173

**Published:** 2026-09-09

**Authors:** Xueying Liu, Linteng Chen, Aiming Chen, Xian-Yang Luo

**Affiliations:** 1 Department of Otolaryngology Head and Neck Surgery, The First Affiliated Hospital of Xiamen University, Xiamen, China; 2 Xiamen Humanity Hospital, Department of Gastroenterology, Xiamen, Fujian, China

**Keywords:** immune checkpoint inhibitor, larynx−preserving surgery rate, locally advanced hypopharyngeal cancer, major pathological response, neoadjuvant therapy, progression-free survival

## Abstract

**Objective:**

To evaluate the efficacy and safety of neoadjuvant PD-1 inhibitor combined with chemotherapy (nab-paclitaxel + cisplatin) in patients with locally advanced hypopharyngeal squamous cell carcinoma.

**Methods:**

A retrospective analysis was conducted on 75 patients with locally advanced (stage III-IVA) hypopharyngeal cancer diagnosed at the First Affiliated Hospital of Xiamen University between January 2021 and May 2023. All patients received at least 2 cycles of neoadjuvant immunochemotherapy followed by radical surgery and adjuvant radiotherapy. Objective response rate (ORR), major pathological response (MPR), pathological complete response (pCR), larynx-preserving surgery rate, progression-free survival (PFS), and safety were evaluated.

**Results:**

Among 75 patients, the ORR was 77.3% (58/75), MPR rate was 78.7% (59/75), standard pCR rate (primary + lymph nodes) was 17.3% (13/75), primary tumor pCR rate was 26.7% (20/75), and larynx-preserving surgery rate was 86.7% (65/75). With a median follow-up of 26 months, the 12-month and 24-month overall survival rates were 93.3% and 76.0%, respectively, and the PFS rates were 92.0% and 76.0%, respectively. The MPR group demonstrated significantly superior PFS compared to the non-MPR group (HR = 0.052, 95% CI: 0.019-0.140, *P* < 0.001), with 24-month PFS rates of 89.7% and 12.5%, respectively. The ORR group also showed significantly superior PFS compared to the non-ORR group (HR = 0.29, 95% CI: 0.13-0.66, *P* = 0.003), with 24-month PFS rates of 86.2% and 41.2%, respectively. Concordance between radiological and pathological assessments was moderate (κ=0.417, *P* < 0.001). The safety profile was favorable, with no grade 3–5 treatment-related adverse events observed.

**Conclusion:**

Neoadjuvant PD-1 inhibitor combined with chemotherapy induces significant radiological and pathological responses in patients with locally advanced hypopharyngeal cancer, effectively improving larynx-preserving surgery rate and progression-free survival with a favorable safety profile. Pathological assessment (particularly MPR) may provide complementary prognostic information to radiological assessment.This regimen demonstrates promising short-term efficacy and larynx preservation benefits, warranting further prospective validation.

## 1 Introduction

Locally advanced squamous cell carcinoma of the head and neck (LA-SCCHN) is the sixth most common malignancy worldwide, with over 700, 000 new cases diagnosed annually and a five-year overall survival rate of less than 40%, demonstrating significant clinical aggressiveness and lethality (1).

Hypopharyngeal squamous cell carcinoma (HPSCC) is the most aggressive subtype of head and neck malignancies, with marked racial disparities in incidence and significant survival differences across populations (2). Locally advanced cases (stage III-IVB) account for a substantial proportion of this disease, and the five-year overall survival rate has consistently remained between 28% and 35% (3, 4). Traditional treatment for locally advanced hypopharyngeal cancer often requires total laryngectomy, which significantly impairs patients’ quality of life (5). Despite multidisciplinary comprehensive treatment—including radical surgery, radiotherapy, and platinum-based concurrent chemoradiotherapy—approximately 50% of patients still experience local recurrence or distant metastasis, resulting in a median overall survival of less than 12 months (6). This challenging clinical situation underscores the urgent need to explore novel therapeutic strategies to improve outcomes.

In recent years, the application of neoadjuvant immunotherapy has expanded from clinical trials to clinical practice exploration in locally advanced squamous cell carcinoma of the head and neck. Previous studies have demonstrated that immune checkpoint inhibitors (ICIs) can induce significant pathological regression, offering a novel strategy to improve patient outcomes (7). ICIs, which reshape the tumor microenvironment by blocking the PD-1/PD-L1 signaling axis, have emerged as a key driver of paradigm shifts in solid tumor therapy (8, 9). The application of biomarkers beyond traditional ones—such as programmed death ligand 1 (PD-L1) and cytotoxic T-lymphocyte-associated antigen 4 (CTLA-4)—has enabled personalized immunotherapy for patients. Among these, the PD-L1 combined positive score (CPS) is a key biomarker that helps predict patient responses to immune checkpoint inhibitors. The phase III KEYNOTE-048 trial demonstrated that pembrolizumab monotherapy prolonged median overall survival (mOS) by 4.2 months compared with the EXTREME regimen (14.9 vs 10.7 months; HR = 0.61) in patients with recurrent/metastatic LA-SCCHN and a PD-L1 CPS ≥20 (10). More recently, the phase III KEYNOTE-689 trial established perioperative pembrolizumab as a new standard of care for resectable locally advanced HNSCC, demonstrating significant improvements in event-free survival (HR = 0.70, *P* = 0.0014) and achieving a 9.4% major pathological response rate (11).These findings provide a solid theoretical rationale and a strong clinical impetus for moving immunotherapy earlier to the neoadjuvant setting with curative intent in locally advanced disease.

The present study aimed to evaluate the efficacy of neoadjuvant immunochemotherapy in locally advanced hypopharyngeal squamous cell carcinoma. We systematically assessed the depth of pathological response, overall survival, progression-free survival, and larynx preservation outcomes induced by this regimen. Through these analyses, we sought to elucidate the clinical potential of immunotherapy in the management of this high-risk disease and to explore directions for future optimization.

## 2 Materials and methods

### 2.1 Study population

**Figure 1:**
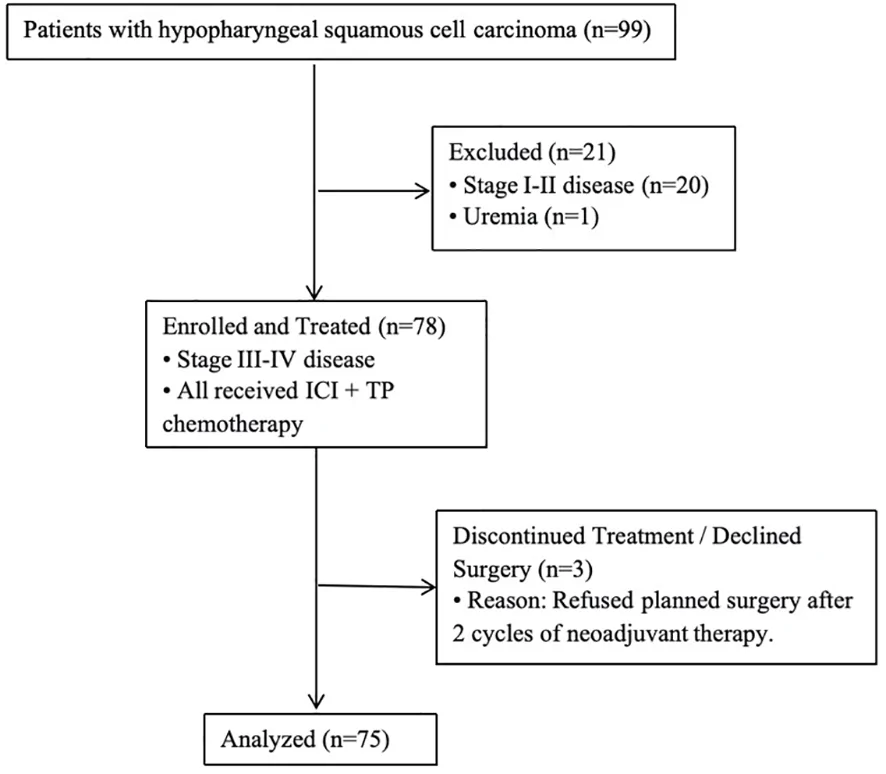
Incorporate the patient flowchart.

This retrospective study enrolled patients diagnosed with locally advanced (stage III–IVA) hypopharyngeal cancer at the First Affiliated Hospital of Xiamen University between January 2021 and May 2023. The enrollment process is illustrated in **Figure 1**. During the study period, all consecutive patients with hypopharyngeal squamous cell carcinoma who presented to our institution and received neoadjuvant therapy were screened for eligibility. **Figure 1** presents the complete workflow of patient screening, enrollment, treatment, and final analysis.A total of 75 patients were ultimately included (**Figure 1**), with ages ranging from 37 to 75 years. The cohort comprised 73 males (97.3%) and 2 females (2.7%).

Inclusion criteria:(1) Patients with pathologically confirmed stage III–IVA hypopharyngeal carcinoma according to the AJCC 8th edition staging system;(2)PD-L1 combined positive score (CPS) assessed prior to treatment;(3)Receipt of at least 2 cycles of neoadjuvant therapy [immune checkpoint inhibitors (ICIs), including tislelizumab, pembrolizumab, camrelizumab, or nivolumab (200 mg intravenously every 3 weeks), in combination with nab-paclitaxel (260 mg/m²) plus cisplatin (75 mg/m²)], followed by surgical resection;(4)Availability of complete imaging and survival follow-up data, with follow-up censored at the date of death or May 2025, whichever occurred first. PD-L1 expression assessment: PD-L1 CPS was assessed using the 22C3 pharmDx assay on the Dako Autostainer Link 48 platform, using formalin-fixed paraffin-embedded biopsy specimens obtained at diagnosis. CPS was defined as the number of PD-L1-positive cells (tumor cells, lymphocytes, macrophages) divided by total viable tumor cells × 100. CPS scoring was independently performed by two experienced pathologists who were blinded to clinical outcomes; discrepancies were resolved by consensus. A CPS threshold of ≥ 20 was used to define PD-L1 high expression, based on the KEYNOTE-048 trial (10).

Exclusion criteria: (1) Prior exposure to anti-PD-1/PD-L1 or CTLA-4 inhibitor therapy; (2) Presence of surgical contraindications, including cardiac dysfunction (LVEF < 50%), hepatic impairment (Child-Pugh class B or C), or renal insufficiency (eGFR < 60 mL/min/1.73 m²); (3) Presence of distant metastasis (M1) or locally recurrent disease; (4) History of hypersensitivity to platinum-based or taxane agents. This study was approved by the Ethics Committee of the First Affiliated Hospital of Xiamen University. Written informed consent was obtained from all patients prior to treatment.

### 2.2 Treatment

All patients were evaluated by a multidisciplinary team (MDT) and subsequently received neoadjuvant immunochemotherapy consisting of immune checkpoint inhibitors (ICIs) combined with nab-paclitaxel (260 mg/m²) plus cisplatin (75 mg/m²), administered in 21-day cycles for a minimum of 2 cycles. Four PD-1 inhibitors—tislelizumab, pembrolizumab, camrelizumab, and nivolumab—were used in this study, all at the standard flat dose of 200 mg intravenously every 3 weeks. The choice among these agents was primarily determined by patient preference and economic considerations, as all were commercially available with different reimbursement policies during the study period. These agents share the same mechanism of action via PD-1/PD-L1 pathway blockade and have demonstrated comparable efficacy and safety profiles in head and neck squamous cell carcinoma; therefore, they were analyzed as a single immunochemotherapy group. The response rates appeared similar across the four agents, although formal subgroup comparisons were precluded by the small sample size in the nivolumab subgroup (n=3).

Radical surgery was performed 4 weeks after the completion of neoadjuvant therapy, with the extent of resection individualized based on tumor response according to RECIST 1.1 criteria. Primary tumor management included transoral low-temperature plasma resection, partial laryngectomy, or total laryngectomy. Neck management was tailored according to initial staging and post-treatment response, with either elective or therapeutic neck dissection performed as indicated (**Table 1**). Postoperatively, all patients received adjuvant radiotherapy (60–66 Gy in 30–33 fractions to the tumor bed and 54–60 Gy to the neck). Concurrent platinum-based chemotherapy was administered to those presenting with high-risk pathological features, including extranodal extension, multiple lymph node metastases, or positive margins.

**Table 1:** Patient's surgical condition.

Surgical procedure	Hypopharyngeal cancer
TL+NLND	10
PL+NLND	7
TORS+NLND	58

TL, total laryngectomy; PL, partial laryngectomy; TORS, transoral resection of primary lesion; NLND, neck lymph node dissection.

### 2.3 Clinical efficacy assessment

Objective response rate (ORR) was evaluated according to RECIST 1.1 criteria, with tumor responses categorized as complete response (CR), partial response (PR), stable disease (SD), and progressive disease (PD). ORR was defined as the proportion of patients achieving CR or PR.

Progression-free survival (PFS) was defined as the time from the initiation of treatment to disease progression or death from any cause, recorded in months. To avoid immortal time bias, since MPR is assessed post-surgery, a landmark analysis was conducted. The landmark time point was set at the date of radical surgery. Progression-free survival (PFS) in the MPR versus non-MPR comparison was therefore redefined as the interval from the date of surgery to disease progression or death. Patients who experienced disease progression or died before surgery were excluded from this specific landmark comparison, as they did not have a pathological response assessment available.

Overall survival (OS) was defined as the time from the initiation of treatment to death from any cause, recorded in months.

Pathological response was assessed using the following criteria: (1) pathological complete response (pCR): no viable tumor cells in either the primary tumor or resected lymph nodes; (2) major pathological response (MPR): ≤10% viable tumor cells remaining in the primary tumor; and (3) incomplete pathological response (IPR): >10% viable tumor cells remaining in the primary tumor.

Larynx-preserving surgery rate was defined as the proportion of patients who did not undergo total laryngectomy. It is important to note that this metric reflects surgical organ preservation and does not capture long-term functional outcomes such as swallowing, voice quality, or permanent tracheostomy dependence.

Postoperative pathological specimens were independently evaluated by two experienced pathologists who were blinded to the radiological findings. Residual viable tumor cells were quantified as the percentage of viable tumor area relative to the total tumor bed area in the resected primary tumor specimen. In cases of disagreement, consensus was reached through discussion. Radiological assessments (contrast-enhanced CT or MRI) were performed 4 weeks after completion of neoadjuvant therapy (i.e., immediately prior to surgery) and were independently reviewed by two radiologists according to RECIST 1.1 criteria, who were also blinded to pathological outcomes.

### 2.4 Adverse reactions

Adverse events were recorded throughout the treatment period and graded according to the Common Terminology Criteria for Adverse Events (CTCAE) version 5.0. The causality of adverse events was assessed by the treating physicians. Immune-related adverse events (irAEs) were managed according to established institutional guidelines, including the use of antihistamines, topical or systemic corticosteroids, and temporary treatment interruptions when clinically indicated.

### 2.5 Statistical analysis

Statistical analyses were performed using SPSS 26.0 (IBM Corp., Armonk, NY, USA) and R software (version 4.2.1, R Foundation for Statistical Computing, Vienna, Austria). A two-sided *P* < 0.05 was considered statistically significant. This study was a retrospective analysis of consecutive eligible patients; therefore, no formal sample size calculation was conducted. Cohen’s Kappa coefficient was adopted to evaluate the concordance between radiological response and major pathological response (MPR). Given the limited number of disease progression events (n = 2) in this retrospective study, multivariate Cox regression analysis was not performed to prevent model overfitting. Survival curves were generated via the Kaplan–Meier method, and intergroup comparisons were carried out using the log-rank test. Univariate Cox regression was used to screen potential prognostic factors, and hazard ratios (HRs) together with 95% confidence intervals (CIs) were presented. The reporting of this study follows the STROBE guidelines for observational studies; the completed checklist is provided in **Table 4**.

## 3 Results

### 3.1 Patient baseline characteristics

A total of 75 patients with locally advanced hypopharyngeal cancer who met the eligibility criteria were ultimately enrolled in this study. The median age was 62 years (range: 37–75 years), and the cohort comprised 73 males (97.3%) and 2 females (2.7%). According to the AJCC 8th edition staging system, 27 patients (36.0%) were classified as stage III and 48 patients (64.0%) as stage IVA. Regarding the choice of immune checkpoint inhibitors, 41 patients received tislelizumab, 17 received pembrolizumab, 14 received camrelizumab, and 3 received nivolumab. The PD-L1 combined positive score (CPS) was ≥ 20 in 30 patients and < 20 in 45 patients. In terms of surgical management, 65 patients underwent larynx-preserving surgery, while 10 patients underwent total laryngectomy (**Table 2**).

**Table 2:** Baseline characteristics of patients.

Variable	N%	ORR		LPS		MPR	
		n(%)	P-value2	n(%)	P-value2	n(%)	P-value2
Age at diagnosis,years							
Range	37-75						
Median							
Gender			>0.999		>0.999		0.383
Female	2(2.7%)	2(3.4%)		2(3.1%)		1(1.7%)	
Male	73(97.3%)	56(96.6%)		63(96.9%)		58(98.3%)	
Smoking			0.367		0.272		0.341
Yes	55(73.3%)	44(75.9%)		46(70.8%)		45(76.3%)	
No	20(26.7%)	14(24.1%)		19(29.2%)		14(23.7%)	
Drinking			0.395		0.154		0.942
Yes	51(68%)	38(65.5%)		42(64.6%)		40(67.8%)	
No	24(32%)	20(34.5%)		23(35.4%)		19(32.2%)	
TNM-stage			0.223		0.084		0.105
III	27(36%)	23(39.7%)		26(40%)		24(40.7%)	
IV	48(64%)	35(60.3%)		39(60%)		35(59.3%)	
PD-1 inhibitor			0.095		>0.999		0.645
Camrelizumab	14(18.7%)	13(22.4%)		12(18.5%)		11(18.6%)	
Nivolumab	3(4%)	3(5.2%)		3(4.6%)		2(3.4%)	
Pembrolizumab	17(22.7%)	15(25.9%)		15(23.1%)		15(25.4%)	
Tislelizumab	41(54.7%)	27(46.6%)		35(53.8%)		31(52.5%)	
CPS			0.115		0.005		0.011
<20	45(60%)	32(55.2%)		35(53.8%)		31(52.5%)	
≥20	30(40%)	26(44.8%)		30(46.2%)		28(47.5%)	
Response rate			–		–		–
CR	9(12%)	–		9(13.8%)		9(15.3%)	
PR	49(65.3%)	–		45(69.2%)		42(71.2%)	
SD	17(22.7%)	–		11(16.9%)		8(13.6%)	
Status			–		–		–
Alive	52(69.3%)	45(77.6%)		52(80%)		50(84.7%)	
Progressed/Death	23(30.7%)	13(22.4%)		13(20%)		9(15.3%)	

CPS, combined positive score; T, tumor; N, nodal; M, metastatic; PD-1, programmed death 1; ORR, objective response rate; MPR, major pathological response; LPS, Larynx-preserving surgery rate; CR, complete response; PR, partial response; SD, stable disease.

Three patients received three cycles of neoadjuvant therapy due to the COVID-19 pandemic, and two patients received three cycles for personal reasons. Another two patients received four cycles due to poor response to the initial treatment. The remaining 68 patients all received two cycles of neoadjuvant therapy.

### 3.2 Efficacy of neoadjuvant therapy

Based on RECIST 1.1 criteria, the radiological responses after neoadjuvant therapy were as follows: 9 patients (12.0%) achieved complete response (CR), 49 patients (65.3%) achieved partial response (PR), and 17 patients (22.7%) had stable disease (SD). No progressive disease (PD) was observed. The objective response rate (ORR) was 77.3%.

Pathological assessment further confirmed substantial tumor regression. Among the 75 patients, major pathological response (MPR) was achieved in 59 patients (59/75, 78.7%), including 13 patients (13/75, 17.3%) with pathological complete response (pCR). When assessed in the primary tumor alone (regardless of lymph node status), the primary tumor pCR rate was 26.7% (20/75). Incomplete pathological response (IPR) was observed in 16 patients (16/75, 21.3%).

The overall larynx-preserving surgery rate was 86.7% (65/75). Notably, the proportion of patients undergoing total laryngectomy was highest in the IPR group (56%), which was significantly higher than that in the MPR group (2%).

### 3.3 Concordance between radiological and pathological responses

**Figure 2:**
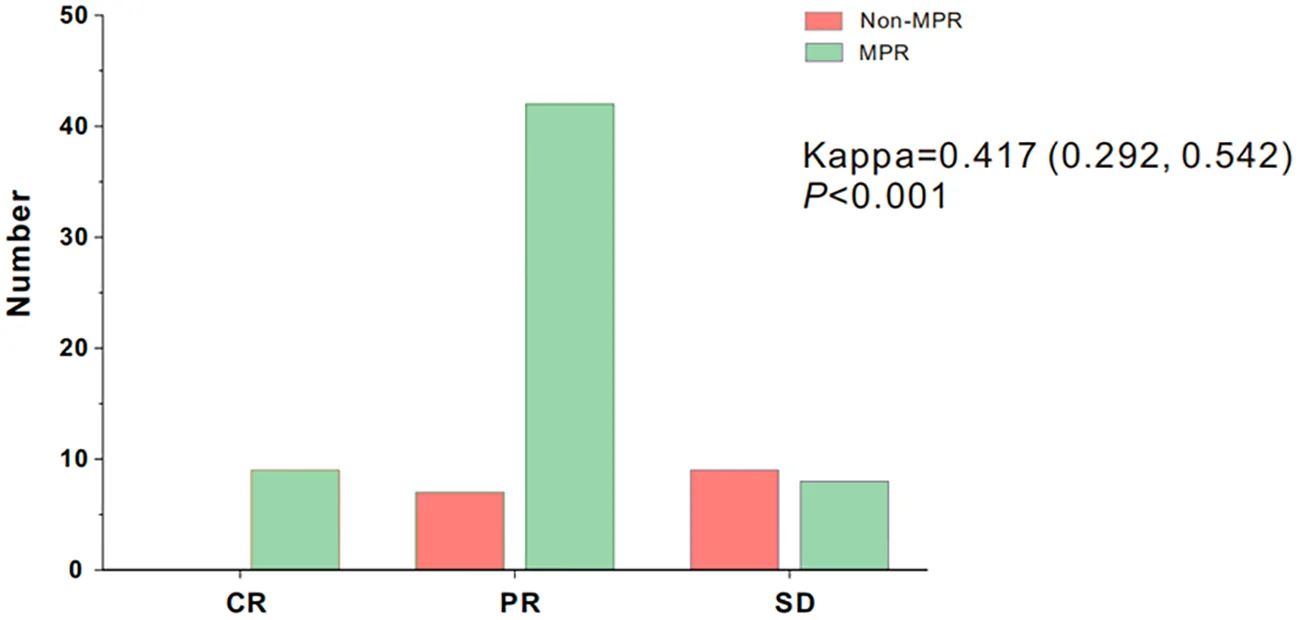
The correlation between pathological response and radiological response.

Using postoperative pathological assessment as the gold standard, the concordance between radiological objective response (ORR) and major pathological response (MPR) was analyzed. The Cohen’s Kappa coefficient was 0.417 (95% CI: 0.292–0.542, *P* < 0.001), indicating moderate agreement between radiological and pathological assessments (**Figure 2**).

### 3.4 Survival analysis

Survival analyses were performed for all patients with a median follow-up of 26.0 months. By the data cutoff (May 2025), 23 deaths (OS events) had been observed among the 75 patients. Of these, only 2 patients died of hypopharyngeal cancer; the remaining 21 deaths were attributed to other causes (including cardiovascular events, other malignancies, and non-cancer-related conditions). No disease progression was documented in these 21 patients. According to the PFS definition (disease progression or death from any cause), the total number of PFS events was 23.

#### 3.4.1 Overall survival outcomes

**Figure 3:**
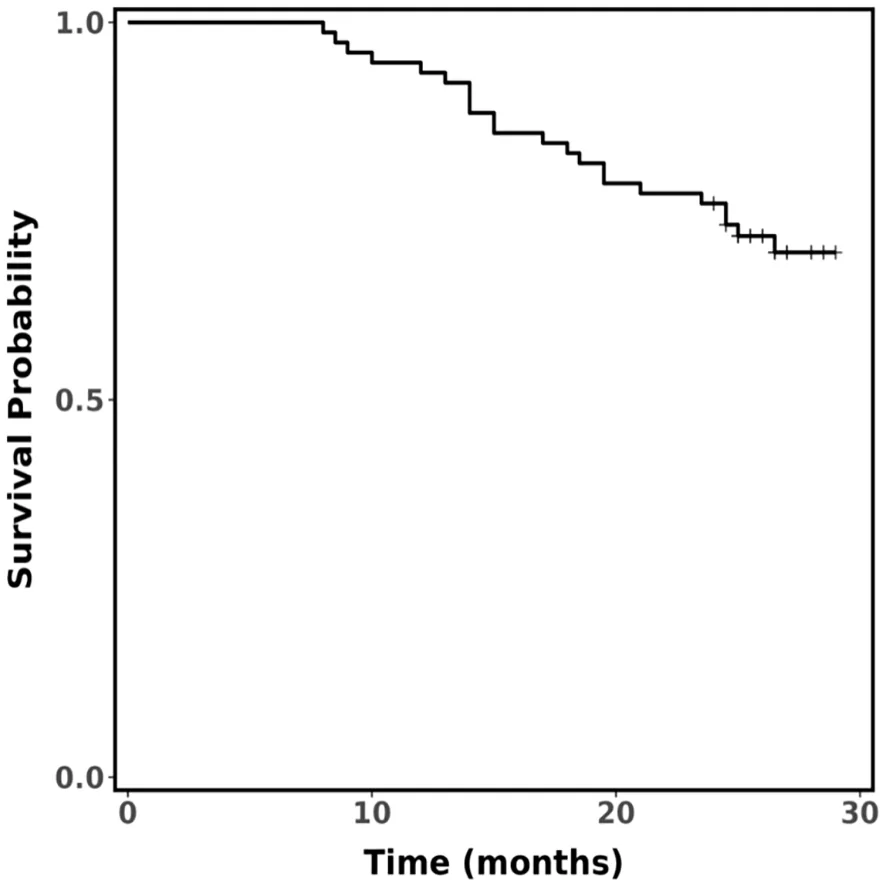
Overall survival curve of the entire cohort.

**Figure 4:**
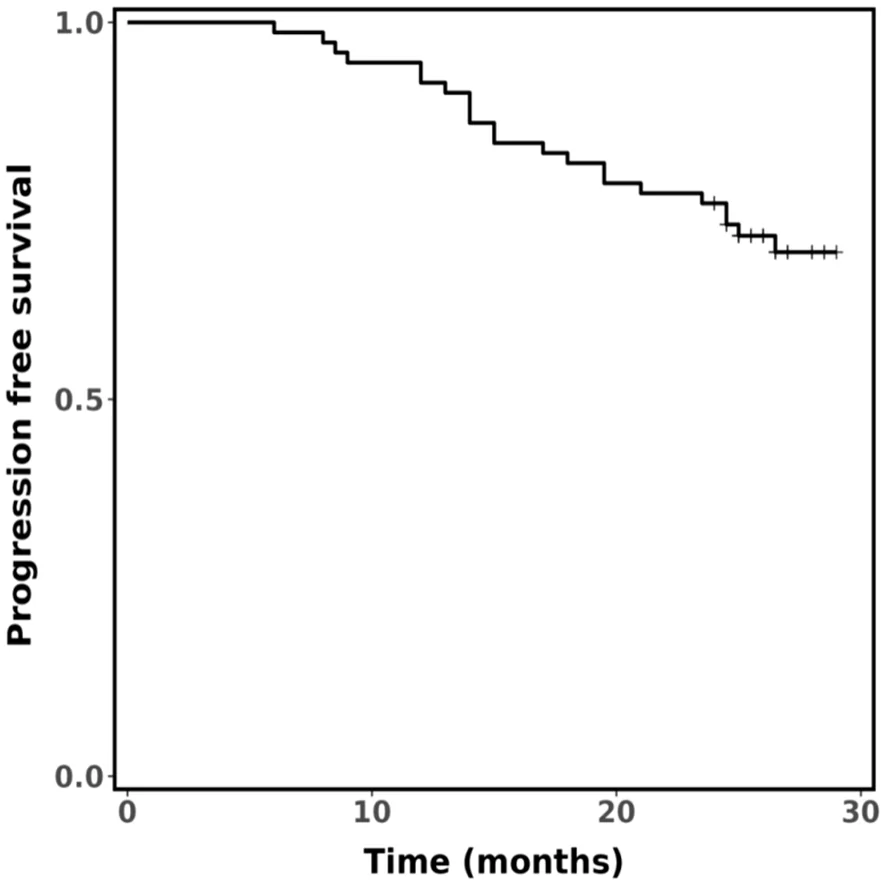
Progression-free survival curve of the entire cohort.

Kaplan–Meier analysis showed that the 12-month and 24-month overall survival (OS) rates for the entire cohort were 93.3% (95% CI: 87.9%–99.2%) and 76.0% (95% CI: 66.9%–86.3%), respectively (**Figure 3**). The 12-month and 24-month progression-free survival (PFS) rates were 92.0% (95% CI: 86.1%–98.4%) and 76.0% (95% CI: 66.9%–86.3%), respectively (**Figure 4**).

As the survival curves did not drop below 50% during the observation period, the median OS and median PFS were not reached (i.e., not estimable). These findings suggest that longer follow-up is warranted to determine the long-term survival benefits of this regimen.

#### 3.4.2 Survival analysis stratified by objective response status

Patients were divided into an objective response group (ORR group, n = 58, 77.3%) and a non-objective response group (non-ORR group, n = 17, 22.7%) according to RECIST 1.1 criteria, and progression-free survival (PFS) was compared between the two groups.

**Figure 5:**
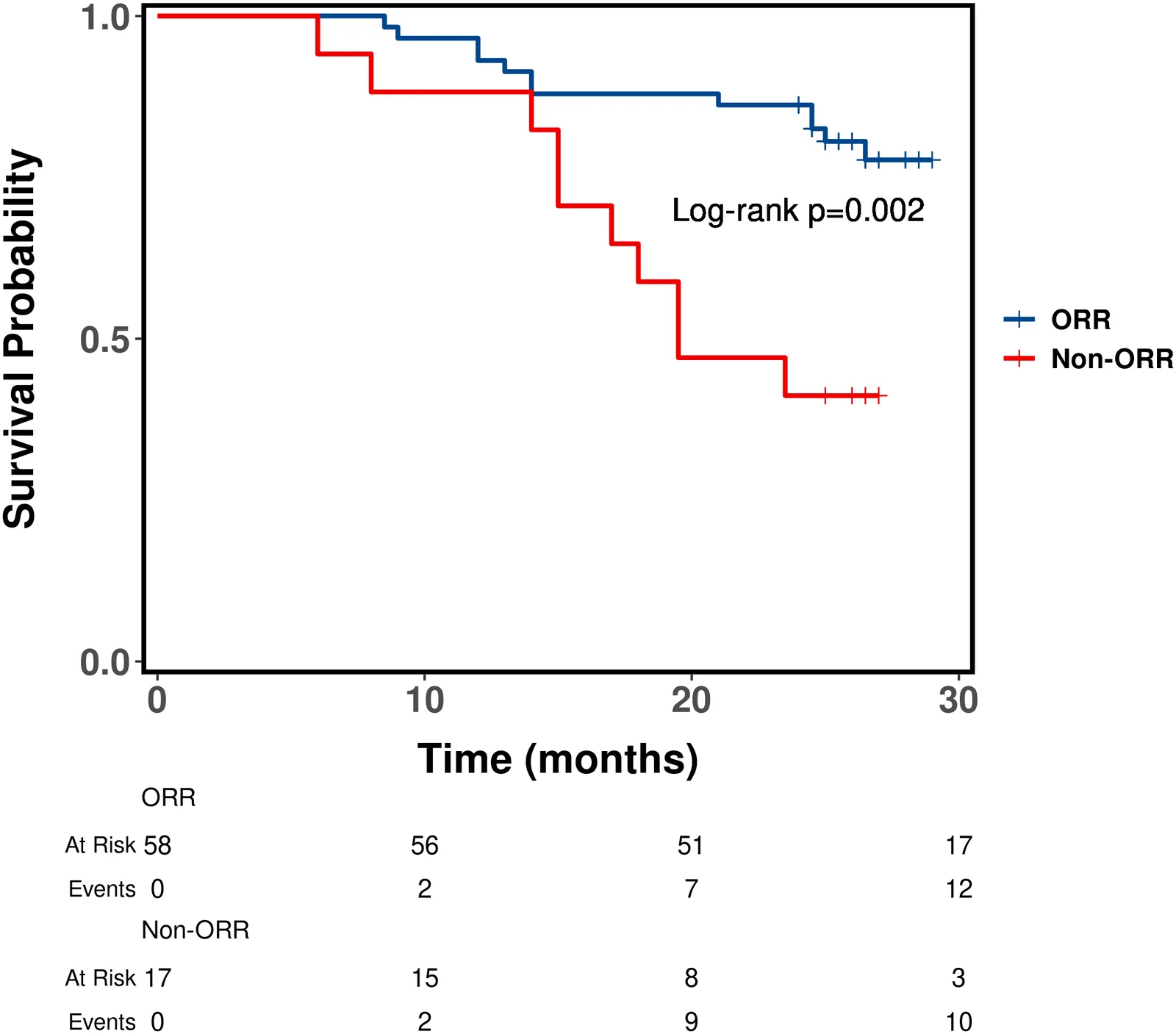
Progression-free survival stratified by objective response rate (ORR).

Kaplan–Meier curves demonstrated that the ORR group had significantly superior PFS compared with the non-ORR group, with a statistically significant difference (log-rank test, *P* = 0.002) (**Figure 5**). The median PFS was not reached in the ORR group, whereas it was 19.5 months (95% CI: 17.0 – not reached) in the non-ORR group. The 12-month and 24-month PFS rates were 93.1% (95% CI: 86.8%–99.9%) and 86.2% (95% CI: 77.8%–95.6%) in the ORR group, compared with 88.2% (95% CI: 74.2%–100.0%) and 41.2% (95% CI: 23.3%–72.7%) in the non-ORR group, respectively.

#### 3.4.3 Survival analysis stratified by pathological response depth

This study further analyzed the impact of major pathological response (MPR) on progression-free survival (PFS) in patients with locally advanced hypopharyngeal cancer. Based on postoperative pathological assessment, patients were divided into the MPR group (≤ 10% viable tumor cells in the primary tumor, n = 59, 78.7%) and the non-MPR group (> 10% viable tumor cells, n = 16, 21.3%), and survival outcomes were compared between the two groups.

**Figure 6:**
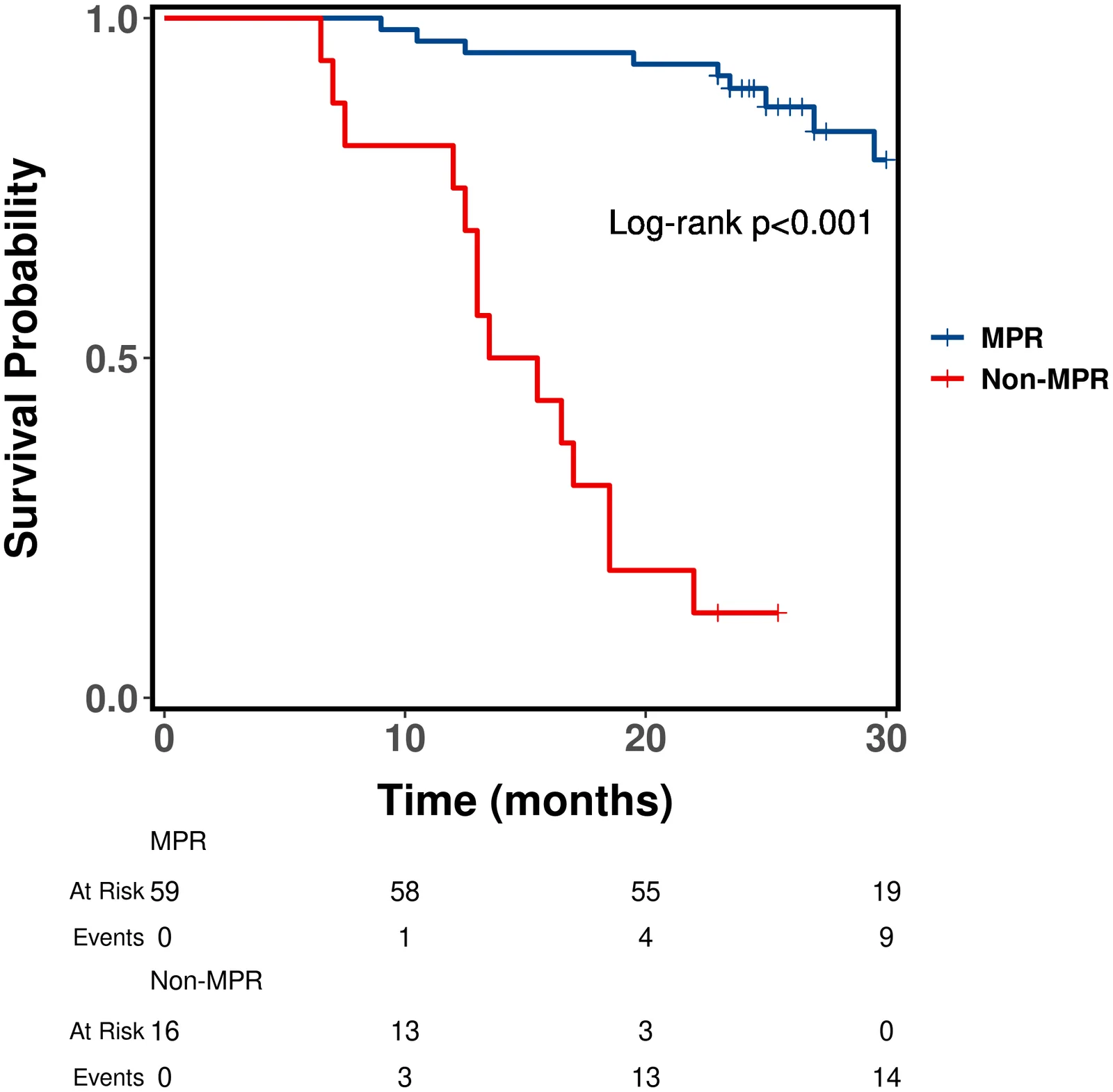
Progression-free survival stratified by major pathological response (MPR).

Note: The PFS analysis for ORR used treatment initiation as the time origin, whereas the MPR analysis used surgery date as the time origin (landmark analysis) to avoid immortal time bias. Therefore, the HRs for ORR and MPR are not directly comparable.

Kaplan–Meier analysis demonstrated that the MPR group had significantly superior PFS compared with the non-MPR group, with a highly significant statistical difference (log-rank test, *P* < 0.001) (**Figure 6**). The median PFS was not reached in the MPR group, whereas it was 14.5 months (95% CI: 12.5–22.0 months) in the non-MPR group. The 12-month and 24-month PFS rates were 96.6% (95% CI: 92.1%–100.0%) and 89.7% (95% CI: 82.1%–97.9%) in the MPR group, compared with 75.0% (95% CI: 56.5%–99.5%) and 12.5% (95% CI: 3.4%–45.7%) in the non-MPR group, respectively.

### 3.5 Safety assessment

The safety profile of the neoadjuvant immunochemotherapy regimen was systematically evaluated in this study. Among all 75 patients, no grade 3–5 treatment-related adverse events (TRAEs) were observed. All patients completed at least 2 cycles of neoadjuvant therapy; no treatment delays, dose reductions, or treatment discontinuations were required due to adverse events.

The most common grade 1–2 adverse events included alopecia (100%), rash/pruritus (60%), fatigue (40%), and nausea/vomiting (33.3%) (**Table 3**). Alopecia was observed in all patients (100%), which is a well-recognized and expected adverse event associated with taxane-based chemotherapy.All grade 1–2 adverse events were managed with symptomatic treatment, including antihistamines for rash/pruritus and antiemetics for nausea/vomiting.Routine prophylactic pegylated recombinant human granulocyte colony-stimulating factor (PEG-rhG-CSF) was administered to all patients receiving chemotherapy, and no grade 3–5 myelosuppression was observed.

**Table 3:** The relevant adverse reactions of PD-1 inhibitors combined with neoadjuvant chemotherapy.

Adverse reactions	Grade 1-2 (%)	Grade 3	Grade 4	Grade 5
Leukopenia	9(12%)	0	0	0
Hypokalemia	5(6.7%)	0	0	0
Hypocalcemia	3(4%)	0	0	0
Anemia	10(13.3%)	0	0	0
Rash/Pruritus	45(60%)	0	0	0
Fatigue	30(40%)	0	0	0
Nausea/Vomiting	25(33.3%)	0	0	0
Diarrhea	5(6.7%)	0	0	0
Thyroid dysfunction	6(8%)	0	0	0
Alopecia	75(100%)	0	0	0
Hepatotoxicity	17(22.7%)	0	0	0
Nephrotoxicity	8(10.7%)	0	0	0

**Table 4:** STROBE Statement checklist for the present study.

Section	Item No.	STROBE Item Description	Location in Manuscript	Fully Reported (Yes/No/N/A)	Comments
Title and Abstract	1a	Indicate study design with standard term in title or abstract	Title (A Retrospective Analysis); Abstract Methods paragraph	Yes	The title explicitly labels this as a retrospective observational study
	1b	Provide balanced, informative summary of methods and key results in abstract	Entire Abstract section	Yes	Abstract covers objective, patient population, treatment regimen, ORR, MPR, larynx-preserving rate, survival endpoints, safety and conclusions
Introduction	2	Explain scientific background and rationale for the investigation	Section 1 Introduction	Yes	Describes high aggressiveness of hypopharyngeal carcinoma, poor survival of traditional therapy, and rationale for neoadjuvant immunochemotherapy supported by KEYNOTE-048
	3	State specific research objectives and pre-specified hypotheses	Last paragraph of Section 1 Introduction	Yes	Clear aims: evaluate pathological response, survival outcomes, larynx preservation and clinical value of neoadjuvant immunochemotherapy
Methods	4	Present core elements of study design early in manuscript	Opening of Section 2.1 Study Population	Yes	Clearly states this is a retrospective analysis of consecutive eligible patients treated between 2021–2023
	5	Describe setting, locations, recruitment/follow-up/data collection timeframes	Section 2.1 Study Population	Yes	Single center: The First Affiliated Hospital of Xiamen University; recruitment Jan 2021–May 2023; follow-up censored May 2025
	6a (Cohort)	Eligibility criteria, participant source, selection methods, follow-up procedures	Section 2.1 Inclusion & Exclusion criteria; **Figure 1** participant flowchart	Yes	Complete inclusion/exclusion criteria listed; all consecutive hypopharyngeal carcinoma patients screened; standardized follow-up until death or censorship date
	6b (Cohort)	Matching criteria and number of exposed/unexposed subjects for matched cohorts	N/A	N/A	No matching design applied in this study
	7	Define all outcomes, exposures, predictors, confounders, effect modifiers; diagnostic criteria provided if applicable	Section 2.3 Clinical Efficacy Assessment	Yes	Standardized definitions for OS, PFS, ORR, pCR, MPR, IPR, larynx-preserving surgery rate; RECIST 1.1, AJCC 8th staging, CTCAE v5.0 referenced; exposure: PD-1 inhibitor plus nab-paclitaxel + cisplatin; predictors: PD-L1 CPS, pathological response status
	8*	Data sources and detailed measurement methods for each variable; comparability of assessment across groups	Section 2.1 PD-L1 testing; Section 2.3 radiological & pathological evaluation	Yes	PD-L1 CPS tested via 22C3 Dako assay; blinded dual radiologist imaging review; blinded dual pathologist pathological quantification; uniform assessment protocols for all patients
	9	Describe strategies used to mitigate potential bias	Section 2.1 & Statistical Analysis paragraph provided previously	Yes	Blinded radiological/pathological review; landmark analysis to avoid immortal time bias; only patients with complete imaging and follow-up data enrolled; Schoenfeld residual test for proportional hazards assumption
	10	Explain how study sample size was determined	Last paragraph of Section 2.5 Statistical Analysis	Yes	Clearly states no formal prospective sample size calculation was performed because this is a retrospective consecutive case analysis
	11	Describe handling of quantitative variables, grouping cut-offs and rationale	Section 2.1 PD-L1 stratification; Section 2.3 pathological response definition	Yes	PD-L1 CPS cut-off ≥20 referenced to KEYNOTE-048; MPR defined as ≤10% viable tumour cells; survival recorded as continuous monthly time
	12a	All statistical methods, including methods to adjust for confounding	Section 2.5 Statistical Analysis	Yes	SPSS 26.0 and R 4.2.1; two-sided P<0.05 threshold; Cohen’s Kappa; Kaplan–Meier & log-rank test; univariate Cox regression; Schoenfeld residual test; multivariate Cox regression omitted due to limited PFS events to avoid overfitting
	12b	Methods for subgroup analyses and interaction testing	Section 3.4 Survival Analysis	Yes	Pre-specified subgroup comparisons: MPR vs non-MPR; ORR vs non-ORR with log-rank test
	12c	Approaches to address missing data	Section 2.1 Inclusion criteria	Yes	Patients with incomplete imaging, pathological or survival data were excluded at screening stage; no missing data present in final analytical cohort
	12d (Cohort)	Methods to handle loss to follow-up	Section 2.1 Study Population & 3.4 Survival Analysis	Yes	All censored patients handled via standard Kaplan–Meier censorship rules; median follow-up clearly reported
	12e	Any sensitivity analyses performed	Discussion limitations paragraph	No	Sensitivity analysis was not conducted due to limited sample size; this limitation is explicitly acknowledged in Discussion
Results	13*a	Number of participants at every study stage (potentially eligible, screened, eligible, enrolled, followed up, analysed)	Section 3.1 Baseline Characteristics; **Figure 1** flowchart	Yes	Total enrolled n=75; flowchart visualises all screening/exclusion stages; breakdown of neoadjuvant treatment cycles provided
	13*b	Reasons for non-participation at each stage	**Figure 1** participant flowchart	Yes	Flowchart lists clear exclusion causes: incorrect tumour stage, absent PD-L1 testing, distant metastasis, incomplete data etc.
	13*c	Recommend use of participant flow diagram	**Figure 1**	Yes	A full CONSORT-style flow diagram is included
	14*a	Baseline demographic, clinical, exposure and confounder characteristics of participants	Section 3.1 Baseline Characteristics; **Table 2** baseline table	Yes	Age, gender, tumour stage, PD-1 agent subtype, PD-L1 CPS grouping, surgical type and neoadjuvant cycles fully summarised
	14*b	Number of participants with missing data per variable	Section 2.1 Inclusion criteria	Yes	No missing data in the final analytical cohort
	14*c (Cohort)	Summary of follow-up duration (median and total)	Section 3.4 Survival Analysis	Yes	Median follow-up of 26 months; 12-month and 24-month survival rates reported
	15* (Cohort)	Number of outcome events or summary outcome measures over follow-up	Section 3.2 Treatment efficacy; 3.4 Survival; 3.5 Safety	Yes	ORR, MPR, pCR, larynx-preserving rates, survival event counts and adverse event frequencies fully quantified
	16a	Unadjusted effect estimates; adjusted estimates with 95% CIs where applicable; confounders adjusted for	Section 3.4 Survival Analysis	Yes	Univariate Cox HR and 95% CIs reported; multivariate analysis not performed with clear methodological justification
	16b	Cut-off values when continuous variables were categorised	Section 3.1 & 3.2	Yes	All categorical thresholds (CPS ≥20, viable tumour cell 10%) clearly defined
	16c	Translation of relative risk measures to absolute risk for clinically relevant timepoints	Abstract & Section 3.4	Yes	12-month and 24-month absolute OS and PFS percentages reported alongside hazard ratios
	17	Report all additional analyses including subgroups, interactions, sensitivity analyses	Section 3.4	Yes	All pre-planned subgroup survival comparisons fully presented; absent sensitivity analysis noted as limitation
Discussion	18	Summarise key results aligned with study objectives	Opening paragraph of Section 4 Discussion	Yes	Core efficacy, survival, concordance and safety results summarised corresponding to research aims
	19	Discuss study limitations, potential bias and imprecision; direction and magnitude of bias addressed	Limitations subsection of Section 4 Discussion	Yes	Single-centre design, lack of parallel control group, limited sample size, short follow-up and absent laryngeal functional assessment discussed; inherent selection bias of retrospective design explained
	20	Cautious overall interpretation integrating objectives, limitations, multiple testing and published literature	Full Section 4 Discussion	Yes	Results compared against historical induction chemotherapy, concurrent chemoradiotherapy and other neoadjuvant immunotherapy cohorts; conclusions not overstated
	21	Discuss generalisability (external validity) of study findings	Final paragraph of Section 4 Discussion	Yes	Conclusions limited to stage III–IVA resectable hypopharyngeal squamous cell carcinoma; further multicentre prospective trials recommended for broader validation
Other Information	22	Source of funding and role of funders	Acknowledgements, Conflict of Interest & Ethical Statement	Yes	Acknowledgements state no funding received; conflict of interest fully declared; ethics approval and patient consent documented

No perioperative surgical complications such as wound dehiscence or hematoma were observed. Postoperative pulmonary infection occurred in 3 patients, all of whom recovered with antibiotic therapy. Pharyngocutaneous fistula developed in 2 patients during adjuvant radiotherapy, all of which resolved with conservative management (anti-infective therapy and local wound care). None of the patients required surgical intervention for these complications.

## 4 Discussion

In recent years, advances in treatment concepts and techniques have led to significant improvements in the prognosis and survival outcomes of patients with hypopharyngeal squamous cell carcinoma (HPSCC) through multimodal approaches integrating surgery, chemotherapy, targeted therapy, and radiotherapy (3, 12). However, for patients with locally advanced HPSCC (LA-HPSCC), radical surgery often comes at the expense of sacrificing critical anatomical structures and functions (13). Therefore, neoadjuvant therapy aimed at tumor downsizing and organ preservation has become a critical component in the management of these patients. In this study, we retrospectively analyzed 75 patients with stage III–IVA HPSCC who received neoadjuvant immunochemotherapy (immune checkpoint inhibitors combined with nab-paclitaxel/cisplatin) followed by surgery, and evaluated the clinical efficacy and potential influencing factors of this treatment modality.

In this study, neoadjuvant immunochemotherapy was associated with improved short-term survival in patients with hypopharyngeal cancer, with 12-month and 24-month progression-free survival rates of 92% and 76%, respectively, and corresponding overall survival rates of 93.3% and 76%. Compared with historical reports of conventional treatment modalities, the present data exhibited a favorable trend. For instance, in large-scale population-based studies, long-term survival for locally advanced hypopharyngeal cancer receiving standard multimodal therapy has consistently remained below 50% (14). Even with effective organ-preservation strategies such as concurrent chemoradiotherapy, long-term disease-free survival rates have plateaued at approximately 44% (15). These findings suggest that neoadjuvant immunotherapy-based strategies may have the potential to overcome the efficacy limitations of traditional therapeutic approaches.

Furthermore, the neoadjuvant immunochemotherapy regimen in this study was associated with substantial tumor regression, with an objective response rate (ORR) of 77.3% and a larynx-preserving surgery rate of 86.7% (defined as avoidance of total laryngectomy). In a recent retrospective analysis, Chen et al. directly compared the efficacy of neoadjuvant PD-1 inhibitors plus chemotherapy, chemotherapy plus EGFR inhibitors, and chemotherapy alone in locally advanced laryngeal and hypopharyngeal cancer. Their results demonstrated that the neoadjuvant immunochemotherapy regimen significantly improved both the ORR (88.2%, *P* < 0.05) and the organ-preservation rate (97.1%, *P* < 0.05) (16), providing preliminary evidence for the dual advantages of ICI-based neoadjuvant strategies in achieving effective tumor control and laryngeal preservation. Although our study differs in terms of regimen details and patient population, we similarly observed high ORR and larynx-preserving surgery rates. Notably, the objective response rate observed in this study appeared higher than the historical benchmarks reported for conventional induction chemotherapy regimens, such as TPF or TP (17). These converging findings suggest that neoadjuvant immunochemotherapy may represent a promising strategy for achieving deep tumor response and improving the likelihood of larynx-preserving surgery in locally advanced hypopharyngeal cancer. However, it should be noted that our larynx-preserving surgery rate reflects surgical organ preservation rather than comprehensive functional preservation, as data on swallowing, voice outcomes, and permanent tracheostomy were not systematically collected. Future studies should incorporate functional assessments to more fully evaluate laryngeal preservation.

Historical data indicate that in the traditional induction chemotherapy setting, even with the highly effective TPF regimen (docetaxel, cisplatin, and 5-fluorouracil), the pathological complete response (pCR) rate has been quite limited. For instance, in a landmark study of patients with resectable hypopharyngeal cancer, the pCR rate after TPF induction chemotherapy was only 9% (18). In contrast, the standard pCR rate (17.3%) and the primary tumor pCR rate (26.7%) observed in the present study both demonstrate substantial improvement. More recently, a study comparing PD-1 inhibitor plus chemotherapy versus targeted therapy plus chemotherapy reported a significantly higher pCR rate in the immunochemotherapy group (37.5% vs. 7.4%) (19). These findings suggest that immune checkpoint inhibitor-based combination regimens may possess inherent advantages over conventional targeted strategies in inducing deep pathological responses.

In this study, the major pathological response (MPR) rate reached 78.7%, which appears to be a promising finding in the neoadjuvant treatment of locally advanced hypopharyngeal cancer.Compared with conventional TPF regimens, for which the MPR/pCR rate is typically below 20%, the results of the present study suggested a several-fold improvement (18), reflecting substantial clinical benefit and providing favorable conditions for surgeons to achieve both radical tumor resection and laryngeal function preservation.

Furthermore, the survival analysis confirmed that MPR is a powerful predictor of progression-free survival (PFS) in patients with locally advanced hypopharyngeal cancer. Patients in the MPR group exhibited remarkable long-term survival advantages, with a 24-month PFS rate of 89.7% (95% CI: 82.1%–97.9%), whereas the corresponding rate in the non-MPR group was only 12.5% (95% CI: 3.4%–45.7%).

Further analysis revealed that radiological objective response (ORR) was also significantly associated with prognosis (*P* = 0.002), indicating that ORR may serve as a valid prognostic indicator. However, the concordance between radiological and pathological assessments was only moderate (κ = 0.417, *P* < 0.001), highlighting the inherent limitations of the RECIST 1.1 criteria—while these criteria can evaluate changes in tumor size, they cannot distinguish viable tumor from therapy-induced non-viable alterations such as necrosis, cystic degeneration, and fibrosis. Consequently, some patients achieving radiological response may still harbor viable tumor cells of clinical significance, whereas pathological assessment enables direct quantification of residual tumor cellularity. Ning et al. (20) similarly reported that, following neoadjuvant immunochemotherapy for head and neck squamous cell carcinoma, the CR rate assessed by contrast-enhanced CT was only 21.0%, compared with a pathological pCR rate of 42.0% (*P* < 0.001), further supporting the notion that radiological evaluation may underestimate the true depth of pathological response. Collectively, these findings indicate that in the context of neoadjuvant immunochemotherapy, reliance on radiological assessment alone is insufficient for determining treatment efficacy, and postoperative pathological evaluation may provide complementary prognostic information to radiological assessment for identifying deep responses and predicting long-term survival. Future research should focus on developing radiomics or functional imaging models to improve the concordance between non-invasive assessments and pathological reference standards, thereby enabling more accurate early identification of patients most likely to benefit from treatment.

In our study, the potential prognostic value of baseline PD-L1 CPS remains unclear. Therefore, given the limited sample size and the exploratory nature of this retrospective analysis, this finding should be interpreted with caution and warrants further investigation.This finding aligns with the current understanding of the complexity of predictive biomarkers. As highlighted in a comprehensive review of predictive markers for pembrolizumab in head and neck cancer (21), although PD-L1 CPS serves as a key stratification tool in the metastatic setting, its predictive performance is substantially influenced by multiple factors, including tumor heterogeneity, treatment modality, and various components of the tumor immune microenvironment—such as tumor mutational burden (TMB), CD8+ T-cell infiltration, and HPV status (22). Additionally, the sample size of our cohort may have been insufficient to detect relatively modest survival differences between CPS subgroups. Moreover, adjuvant therapies (e.g., radiotherapy) administered after neoadjuvant treatment may have balanced the long-term outcomes across different CPS groups, thereby attenuating the predictive signal of baseline CPS. Therefore, future efforts should focus on developing integrative models that combine multiple biomarkers to more precisely identify the patient population most likely to benefit from neoadjuvant immunotherapy.

Several limitations of this study should be acknowledged. First, as a single-center retrospective analysis without a concurrent control arm, the findings are subject to inherent selection bias and residual confounding. Therefore, the present results should be interpreted as hypothesis-generating rather than confirmatory, and causal inferences cannot be drawn. The observed favorable outcomes warrant validation in future prospective, multicenter randomized controlled trials.Second, the modest sample size may have limited statistical power, particularly for subgroup analyses. Third, the follow-up duration is relatively short, and long-term survival data are still maturing, which precludes a full assessment of the long-term benefits of this regimen. Future large-scale, multicenter, prospective randomized controlled trials are warranted to validate the efficacy and safety of neoadjuvant immunochemotherapy in locally advanced hypopharyngeal cancer and to define its value across biomarker-defined subgroups.

## 5 Conclusions

In conclusion, neoadjuvant PD-1 inhibitor combined with chemotherapy induces significant radiological and pathological responses in patients with locally advanced hypopharyngeal cancer, effectively improves larynx-preserving surgery rate and progression-free survival, and demonstrates favorable short-term efficacy with advantages in laryngeal organ preservation. Future large-scale, prospective randomized controlled trials with extended follow-up are warranted to further validate the definitive survival benefits of this strategy.

## Data Availability

The original contributions presented in the study are included in the article/supplementary material. Further inquiries can be directed to the corresponding authors.
